# Development of Psychological Problems Among Adolescents During School Closures Because of the COVID-19 Lockdown Phase in Italy: A Cross-Sectional Survey

**DOI:** 10.3389/fped.2020.628072

**Published:** 2021-01-22

**Authors:** Susanna Esposito, Nino Giannitto, Antonella Squarcia, Cosimo Neglia, Alberto Argentiero, Paola Minichetti, Nicola Cotugno, Nicola Principi

**Affiliations:** ^1^Pediatric Clinic, Pietro Barilla Children's Hospital, University of Parma, Parma, Italy; ^2^Unit of Emergency Pediatrics, Department of Human Pathology in Adult and Developmental Age “Gaetano Barresi,” University of Messina, Policlinico “G. Martino,” Messina, Italy; ^3^Unit of Neuropsychiatry of Children and Adolescents, Azienda Unità Sanitaria Locale (AUSL) Parma, Parma, Italy; ^4^Academic Department of Pediatrics (DPUO), Research Unit of Congenital and Perinatal Infections, Bambino Gesù Children's Hospital, Rome, Italy; ^5^Università degli Studi di Milano, Milan, Italy

**Keywords:** adolescent, COVID-19, mask, psychological problems, SARS-CoV-2, school

## Abstract

**Background:** Previous studies have shown that during COVID-19 pandemic, mainly due to the imposed lockdown, significant psychological problems had emerged in a significant part of the population, including older children and adolescents. School closure, leading to significant social isolation, was considered one of the most important reasons for pediatric mental health problems. However, how knowledge of COVID-19 related problems, modification of lifestyle and age, gender and severity of COVID-19 pandemic had influenced psychological problems of older children and adolescents has not been detailed. To evaluate these variables, a survey was carried out in Italy.

**Methods:** This cross-sectional survey was carried out by means of an anonymous online questionnaire administered to 2,996 students of secondary and high schools living in Italian Regions with different COVID-19 epidemiology.

**Results:** A total of 2,064 adolescent students (62.8% females; mean age, 15.4 ± 2.1 years), completed and returned the questionnaire. Most of enrolled students showed good knowledge of COVID-19-related problems. School closure was associated with significant modifications of lifestyle and the development of substantial psychological problems in all the study groups, including students living in Regions with lower COVID-19 incidence. However, in some cases, some differences, were evidenced. Sadness was significantly more frequent in females (84%) than males (68.2%; *p* < 0.001) and in the 14–19-year-old age group than the 11–13-year-old age group (79.2% vs. 70.2%; *p* < 0.001). Missing the school community was a significantly more common cause of sadness in girls (26.5% vs. 16.8%; *p* < 0.001), in southern Italy (26.45% vs. 20.2%; *p* < 0.01) and in the 14–19-year-old group (24.2% vs. 14.7%; *p* < 0.001). The multivariate regression analysis showed that male gender was a protective factor against negative feelings (*p* < 0.01), leading to a decrease of 0.63 points in the total negative feelings index. Having a family member or an acquaintance with COVID-19 increased the negative feelings index by 0.1 points (*p* < 0.05).

**Conclusions:** This study shows that school closures because of the COVID-19 pandemic outbreak was associated with significant lifestyle changes in all the students, regardless of age and gender. Despite some differences in some subgroups, the study confirms that school closure can cause relevant mental health problems in older children and adolescents. This must be considered as a reason for the maintenance of all school activities, although in full compliance with the measures to contain the spread of the pandemic.

## Background

Several studies have shown that severe infectious disease outbreaks affecting multiple countries and communities can impact not only physical but also psychological health (1–3). Although positive changes and cognitive restructuring were described in some cases, generally, a series of negative consequences was evidenced. Development of anxiety, fear, somatic symptoms, sleep disturbance, depression, feelings of anger and irritability, grief and loss, post-traumatic stress, and stigmatization due to severe infection disease outbreaks have been repeatedly reported in a significant part of the population. A cross-sectional study carried out in Singapore 16 weeks after the first national outbreak of SARS showed that 22.9 and 25.8% of 415 individuals visiting community health care services had suffered or were suffering from SARS-related psychiatric or posttraumatic morbidities, respectively (4). Similar or even higher rates of acute and long-term psychosocial effects at the individual, community, and international levels were demonstrated during and after the 2009 influenza pandemic and Ebola epidemic (5, 6).

As expected, the novel coronavirus disease 2019 (COVID-19) pandemic has been accompanied by the development of significant psychological problems in a considerable part of the population; however, the incidence and type of psychological reactions have varied significantly according to the characteristics of the involved population and the incidence rate of the disease in the geographic area where the problem has been studied (7, 8). The most vulnerable groups seem to be health-care workers, homeless people, migrant workers, pregnant women, and people who were already mentally ill. Moreover, it has been suggested that other vulnerable populations, such as people leaving in remote or rural areas who face barriers in accessing health care and those living in lower socioeconomic conditions, may develop relevant psychological problems (9). The same seems true for older children and adolescents, who, mainly due to school closures, remain socially isolated and may lose access to all the health services that are delivered via schools, including mental health services (10).

Italy was deeply affected by the present COVID-19 pandemic, but there were differences in the effects between the northern and southern regions. On August 25, 2020, 261,174 laboratory-confirmed severe acute respiratory syndrome coronavirus 2 (SARS-CoV-2) cases and 35,445 deaths were reported to the Ministry of Health in Italy, among which more than 70% were diagnosed in the northern regions (11). Since March 8, school closures until the end of the school year have been recommended in Italy, and lessons have been performed via tele-teaching (11). The impact of school closure in Italian students has not been previously evaluated. In particular, it is not known whether impact on lifestyle and mental health was different in younger compared to older students, in males compared to females and in children living in Regions with different COVID-9 epidemiology. This knowledge seems essential to understand which measures must be put in place in Italy to reduce potential health problems related to COVID-19 among older children and adolescents that do not attend school to limit SARS-CoV-2 diffusion. To make a contribution in this regard a study was performed.

## Methods

### Participants and Procedure

This was a cross-sectional survey with the main aim to evaluate whether school closure during COVID-19 pandemic had caused in older children and adolescents significant modifications of the current lifestyle potentially associated with the development of significant psychological problems. Moreover, the role of some variables (knowledge of COVID-19 characteristics, age, gender, type of school, incidence of COVID-19 in the region where the student was living and presence of one or more relatives among SARS-CoV-2 infected subjects) in the development of psychological problems was tentatively evaluated. To collect data and ensure the confidentiality and reliability of the data an anonymous online questionnaire developed by pediatricians, infectious diseases specialists, child neuropsychiatrists and psychologists was used. Google Forms® was used to collect information from users. The questionnaire was disseminated through schools and private channels. The personal that were data collected were age, sex, type of school and region of residence. Participants accessed the questionnaire through a web link. Data collection took place for a period of <2 weeks (8–21 April 2020) 1 month after the beginning of school closures because of the COVID-19 lockdown. The privacy policy was approved by the Data Protection Office of the University of Parma. Expedited ethics approval was obtained from the Institutional Review Board of the University of Parma, which conforms to the principles embodied in the Declaration of Helsinki, 4 weeks after the World Health Organization (WHO) declared the COVID-19 outbreak to be a pandemic. Both parents and students had to provide their written consent.

The online survey was first disseminated to the parents of students of 2 secondary and 3 high schools with different educational orientations located in Parma. The schools were chosen among those with the highest number of students after the school administrators and the teachers had given their consent to allow the study to be carried out. With the teachers it was established that, on the day of the distribution of the questionnaire, they would explain to the students the meaning of the questionnaire and would clarify any doubts about its completion. To have information on the impact of the pandemic on students who lived in geographical areas less affected by the spread of SARS-CoV-2, the teachers at Parma schools were encouraged to contact some colleagues from Southern Regions, explain the importance of the survey and obtain the cooperation for the distribution of the questionnaire to their students. A total of 7 schools sited in Italian Regions other than Emilia-Romagna, the Region where Parma is sited, was involved in the study. The dissemination of the questionnaire took place in the same way as in Parma. The online form comprised six parts: (1) an introductory part addressed to parents that requested that they accept the privacy policy; (2) an introductory part aimed at students; and (3–6) the questionnaire parts. The authors drew from the measurement instruments of studies on the psychological impacts of SARS and influenza outbreaks that were mainly devoted to the evidence of the development of depression and anxiety (12, 13) and included additional questions related to the COVID-19 outbreak. The structured questionnaire (Table 1) consisted of questions covering several areas: (1) demographic data and school locations, (2) knowledge and concerns about COVID-19, (3) additional information related to COVID-19, (4) the psychological impact of the COVID-19 outbreak, and (5) mental health status.

**Table 1:** Questionnaire presented during the survey.

**Demographic information** 1. Sex 2. Age 3. School type 4. Region of residence **Knowledge of COVID-195**. Do you know what is COVID-19? 6. What tool do you mainly use to find out about COVID-19? 7. Do you think you are at risk of contracting COVID-19? 8. What are the most common symptoms of COVID-19 infection? 9. How is COVID-19 diagnosed? 10. Do you think there is a treatment for COVID-19? 11. Have you been told that you were sick with COVID-19? 12. Have any of your acquaintances contracted COVID-19? If yes, specify who. **Psychological impact of COVID-19 on teenagers** 13. Are you afraid of COVID-19? 14. Since schools have been closed, have you noticed that you are sleeping more or less? 15. If you have been sleeping less or more, explain why. 16. Have you been more agitated since schools have been closed? 17. Since schools have been closed, have you been feeling sad? 18. Do you cry during the day? 19. Do you feel more tired than usual during the day? 20. What do you miss most since schools have been closed? 21. Since schools have been closed, have you been eating more or less?	22. If you have been eating more or less, why do you think this has happened? 23. Since schools have been closed, have been speaking more or less with your parents? 24. Since schools have been closed, have your relationships with your improved, deteriorated or remained the same? 25. Since schools have been closed and the authorities have said to decrease outings and contacts with the others, is there something you have missed? 26. Do you ever talk about COVID-19 with your friends? 27. Would you like to be able to discuss/talk more about COVID-19? 28. Who would you like to talk about COVID-19 with? 29. Since you have been at home, have you taken your temperature due to a fear of having COVID-19? 30. Since you have been home from school, have you been seen by a doctor for COVID-19? 31. Would you like to take a swab test to find out if you have COVID-19? 32. Would you like to provide a blood sample to find out if you have had COVID-19? **Lifestyles** 33. Do you attend school online? 34. Do you go out sometimes? 35. Do you see any of your friends? 36. Since schools have been closed, have you been spending more time playing video games? 37. Do you wash your hands more than before? 38. Do you have a surgical mask?

### Statistical Analysis

The continuous variable “age” was characterized as the mean ± standard deviation, and the categorical variables (gender, region of residence, type of school, age group, and frequency) for each item were characterized by frequencies and percentages. The age groups considered in the analysis were 11–13 and 14–19 years old. The Wilcoxon rank–sum test was used to compare the difference in age between groups. The chi–square test or Fisher's exact test, when the frequency was ≤5, was performed to compare the frequency of answers for each question between groups. The same tests were used to compare the difference in the frequency of a single response between groups. For better assessing impact of pandemic on psychological status of enrolled children a negative feeling index was developed. It was calculated as the sum of the following dichotomous variables: presence of fear, restless, sadness, and variation in the hours of sleep, considered 0 for absence and 1 for presence. A multivariate regression model to assess the association between the negative feelings index and a series of variables, such as age in years, sex, and personal experience of at least one relative or acquaintance with COVID-19. All statistical analyses were performed by using Stata Statistical Software: Release 12 (College Station, TX, USA).

## Results

### General Characteristics of the Study Population

Table 2 summarizes the general characteristics of the study population. A total of 2,064 students (62.8% females; mean age, 15.4 ± 2.1 years), 69% of those initially included in the study, completed and returned the questionnaire. Subjects who did not return the questionnaire were equally distributed among northern and southern students, without differences regarding age, gender and type of school. Among those who returned the questionnaire, 1,290 (62.5%) were residents in northern Italian regions, mainly Lombardy and Emilia-Romagna, while 774 (37.5%) lived in southern regions, mainly Sicily and Puglia. In both northern and southern Italy, females represented approximately two-thirds of the studied population. However, individuals aged 14–19 years and those who were attending high school were significantly more numerous in the northern regions than in the southern regions, where subjects aged 11–13 years and those attending professional and technical schools were more numerous.

**Table 2:** General characteristics of the study population.

	**Northern Italy**	**Southern Italy**	***p*-value**
**N**	1,290	774	
**Sex**			0.317
Females	800 (62.0)	497 (64.21)	
Males	490 (38.0)	277 (35.79)	
**Age, years**	15.3 ± 2.2	15.7 ± 1.8	0.0001
Age groups			<0.0001
11–13 years	287 (22.2)	87 (11.2)	
14–19 years	1,003 (77.8)	687 (88.8)	
**School**			<0.0001
Professional institute	158 (12.2)	222 (28.7)	<0.0001
Technical institute	144 (11.2)	124 (16.0)	0.01
High school-liceo	660 (51.2)	325 (42.0)	<0.0001
Middle school	328 (25.4)	103 (13.3)	<0.0001

*Percentages in parentheses*.

### Knowledge of Covid-19

Table 3 summarizes knowledge about Covid-19 in the study population according to gender, area of residence and age. Most students evidenced the high lethality and lack of effective therapy of COVID-19. The lethality of COVID-19 was significantly more frequently highlighted by females than males (*p* < 0.01), while the lack of an effective therapy for the disease was more frequently reported by older students (58.6 vs. 49.2%; *p* < 0.001), Knowledge about symptoms and diagnosis of COVID-19, and personal risk of infection was generally good, without significant differences according to sex, age, type of school, and geographic area of origin. Mass media were largely used by all the students to access information on COVID-19. However, while mass media were the most frequently used tools by older students, parents were the source of information most frequently consulted by children under 13 years of age. Personal experience with a relative or an acquaintance affected by COVID-19 was significantly more frequently reported by northern students than by southern students (53.7 vs. 11.6%; *p* < 0.001).

**Table 3:** Knowledge about COVID-19 in the study population according to gender, area of residence, and age.

	**% Females**	**% Males**	***p*-value**	**% North**	**% South**	***p*-value**	**% age 11–13 yrs *N* = 374**	**% age 14–19 yrs *N* = 1,690**	***p*-value**
**Do you know what COVID-19 is?**			0.001			0.077			0.309
A virus that can only rarely be lethal	3.3	5.9		5.0	3.0		4.3	4.3	
A lethal virus in all ages but especially in the elderly and chronically ill	85.8	80.6		83.8	84.0		83.7	83.9	
A lethal virus only in the elderly and chronically ill	4.5	7.6		5.5	5.8		7.2	5.3	
An often lethal virus	6.4	6.0		5.7	7.2		4.8	6.6	
**Which tool did you mainly use to find out about COVID-19?**			0.824			<0.0001			<0.001
Talking to friends	2.0	2.0		2.0	1.9		2.7	1.8	
Talking to my parents	21.7	23.5		23.5	28.0		44.1	17.5	
Online	55.1	54.0		54.0	50.7		40.9	55.3	
In newspapers	44.9	46.0		46.0	49.3		12.3	25.3	
**Do you think you are at risk of contracting COVID-19?**			0.333			0.031			<0.001
No	26.4	30.0		26.8	29.2		31.8	26.8	
Yes, other	5.5	5.1		6.4	3.5		4.8	5.4	
Yes, we are all at risk	56.4	54.4		55.5	55.8		47.1	57.5	
Yes, one of my parents does a risky job	11.8	10.6		11.2	11.5		16.3	10.2	
**What are the most common symptoms in COVID-19 infections?**			0.138			0.317			0.538
Fever, sore throat, and muscle pain	1.9	2.5		2.5	1.6		1.9	2.2	
Fever, sore throat, cold, and breathing difficulties	96.7	94.8		95.8	96.3		95.5	96.1	
Fever, nausea, and diarrhea	0.5	1.0		0.5	1.0		0.8	0.7	
Vomiting, breathing difficulties, and diarrhea	0.9	1.7		1.2	1.2		1.9	1.0	
**How is COVID-19 diagnosed?**			0.241			0.012			0.375
With blood tests	0.7	1.4		1.1	0.8		1.6	0.8	
With an examination on the pharyngeal swab and nose	98.0	97.1		97.1	98.6		96.8	97.9	
With an x-ray	0.8	0.5		1.1	0.0		0.5	0.7	
With a medical examination	0.5	0.9		0.7	0.7		1.1	0.6	
**Do you think there is a therapy against COVID-19?**			0.339			0.277			<0.001
No, I don't	57.9	55.2		57.5	55.8		49.2	58.6	
Yes, for all	23.2	23.2		23.2	23.2		20.9	23.8	
Yes, only for the most serious cases	18.9	21.4		20.3	19.0		29.9	17.6	
**Did they tell you that you got sick from COVID-19?**			0.729			0.161			0.014
No	96.5	96.7		96.1	97.3		98.7	96.1	
Yes	3.6	3.3		3.9	2.7		1.3	3.9	
**Did any of your contact COVID-19?**			0.024			<0.001			0.190
No	60.2	65.2		46.3	88.4		59.1	62.7	
Yes	39.8	34.8		53.7	11.6		40.9	37.3	
									0.190
If yes, specify	40.1	35.2		54.2	11.8				
A relative of mine	10.6	9.3	0.31	15.1	1.9	<0.001	13.1	9.2	0.021
A acquaintance of mine	25.8	21.8	0.038	34.3	7.8	<0.001	24.6	24.3	0.909
A friend of my friends	9.0	8.5	0.678	12.5	2.8	<0.001	7.7	9.1	0.403

### Lifestyle

Table 4 summarizes the questionnaire results about lifestyle during pandemic period. Of the total sample, 65% said they were satisfied with doing school online, without significant differences between the groups. Regarding leaving home, 74.6% said they did not go out, while 11.8% went out only once a week. Among those who responded “I don't go out,” there were statistically significant differences by gender, age group, and region with females, older children, and subjects living in the Southern Regions the subjects with the greater tendency to remain at home. For a greater use of video games than before, a significantly higher frequency was found in males (76%) and the 11–13-year age group (65%) than in females (43%) and the 14–19-year age group (53%).

**Table 4:** Psychological impact of school closure because of COVID-19 lock-down in the study population according to gender, area of residence, and age.

	**% Females**	**% Males**	***p*-value**	**% North**	**% South**	***p*-value**	**% age 11–13**	**% age 14–19**	***p*-value**
**Do you happen to be afraid of COVID-19?**			<0.001			<0.001			<0.001
No	14.6	36.5		21.5	24.8		24.1	22.4	
Yes, I'm afraid my parents will die	15.8	10.8		15.1	12.0		15.2	13.7	
Yes, I'm afraid my grandparents will die	32.0	24.6	<0.001	33.6	22.0	<0.001	36.6	27.6	0.01
Yes, I'm afraid of getting sick and being hospitalized	9.8	10.6		9.9	10.3		9.1	10.3	
Yes, I'm afraid of dying	4.1	2.2		2.3	5.2		4.8	3.1	
Yes, for other resasons	23.8	15.3		17.5	25.7		10.2	22.9	
**Since schools are closed, you noticed**			<0.001			0.058			<0.001
Yuo sleep as before	22.4	29.6		25.7	24.2		23.0	25.6	
You sleep more	34.5	46.8		40.2	37.2		48.4	37.0	
You sleep less (total)	43.1	23.6		34.1	38.6		28.6	37.5	
You sleep less because you struggle to fall asleep	27.4	15.0		22.3	23.6		16.6	24.1	
You sleep less because you have nightmares	2.1	0.4		1.6	1.2		2.4	1.2	
You sleep less because you wake up early	6.6	4.2		4.6	7.6		4.8	5.9	
You sleep less because you wake up often	7.0	4.0		5.7	6.2		4.8	6.1	
**If you sleep less or more, explain why**			<0.001			<0.001			<0.001
I sleep less because I'm afraid that one of my relatives is sick	10.1	3.9		8.3	7.3		8.7	7.8	
I sleep less because I'm afraid of COVID-19	2.9	1.8		2.6	2.3		3.1	2.4	
I sleep less because I'm afraid of getting sick	2.3	0.8		1.4	2.3		1.4	1.9	
I sleep less because I'm afraid of dying	0.7	0.6		0.3	1.3		1.1	0.6	
I sleep less because I am less tired	35.5	24.1		30.2	33.8		21.3	34.1	
I sleep more because I don't have to go to school	26.4	41.2		36.9	22.7		43.9	28.5	
I sleep more because I can't do anything	6.6	8.2		6.8	7.7		7.3	7.1	
I sleep more because I can't meet my friends	5.7	7.1		5.1	8.1		4.2	6.7	
I sleep more because I'm bored	9.8	12.4		8.3	14.5		9.1	11.1	
**Have you been more agitated since schools are closed?**			<0.001			0.203			0.025
No	38.1	57.5		46.1	43.9		45.7	45.2	
Yes, I'm afraid my relatives will get sick	14.0	9.0		13.3	10.3		14.7	11.6	
Yes, I'm afraid of COVID-19	5.1	4.4		4.3	5.8		6.1	4.6	
Yes, I'm afraid of getting sick	1.9	2.4		2.1	1.9		3.2	1.8	
Yes, I'm afraid of dying	0.9	0.3		0.6	0.8		1.1	0.6	
Yes, because I'm afraid of not being able to stay behind the studio	15.4	11.9		13.3	15.4		9.9	15.0	
Yes, because I feel alone and close to friends	24.6	14.6		20.3	21.8		19.2	21.2	
**Since when the schools are closed, have you been feeling sad?**			<0.001			<0.001			<0.001
No	16.0	31.8		22.1	21.5		29.4	20.2	
Yes, because I feel a sense of loneliness	42.5	32.3		39.1	38.1		34.0	39.8	
Yes, because I'm afraid that my relatives will get sick	5.1	3.4		4.9	3.8		4.1	4.4	
Yes, because I'm afraid of COVID-19	1.8	2.7		1.7	2.8		2.1	2.1	
Yes, because I'm afraid of getting sick	0.8	0.5		0.3	1.3		1.1	0.6	
Yes, because I'm afraid of dying	0.8	-		0.5	0.4		0.3	0.5	
Yes, because I miss school as a community	26.5	15.8		20.2	26.4		14.7	24.2	
Yes, because I miss sport	6.7	13.4		11.2	5.8		13.6	8.2	
**Do you happen to cry during the day?**			<0.001			0.953			<0.001
No	51.3	86.6		64.3	64.5		72.7	62.5	
Yes	48.7	13.4		35.7	35.5		27.3	37.5	
**Do you feel more tired than usual during the day?**			<0.001			0.053			<0.001
No	50.1	64.7		54.4	58.8		64.7	54.1	
Yes	49.0	35.3		45.6	41.2		35.3	45.9	
**What did you regret most about closing schools?**			<0.001			0.017			0.004
Don't do lessons the traditional way	13.0	7.2		9.5	13.2		7.7	11.5	
Not being able to run program experiences	16.1	9.3		12.9	14.6		11.0	14.1	
Not being able to go out in the morning	6.9	4.7		6.2	5.9		3.7	6.6	
Don't see school friends	58.1	70.9		65.4	58.8		69.5	61.4	
Nothing	5.8	8.0		6.1	7.5		8.0	6.3	
**Since schools are closed**			<0.001			0.011			0.267
I have more snacks	20.4	19.2		20.8	18.6		22.7	19.3	
I eat as before	28.6	41.6		35.4	30.1		34.2	33.2	
I eat less	15.5	9.6		12.6	14.6		11.0	13.8	
I eat more	35.5	29.6		31.2	36.7		32.1	33.5	
I eat more + I have more snacks	55.9	48.8		52	55.3		54.8	52.8	
**If you eat more or less, why?**			0.001			0.304			0.005
I eat less because I'm less hungry	14.9	13.8		15.3	13.2		12.5	14.9	
I eat less because I have more difficulty digesting	1.6	1.1		1.7	1.0		2.2	1.3	
I eat less because my stomach has closed	6.4	3.6		4.5	7.1		3.9	5.8	
I eat less because I can't go to the canteen	0.3	1.1		0.7	0.4		1.7	0.4	
I eat more because I finally eat at home at lunchtime	5.4	10.2		7.5	6.1		10.0	6.4	
I eat more because I'm more hungry	16.0	20.6		17.1	18.2		22.9	16.4	
I eat more because I am bored	35.8	29.4		33.7	33.5		27.7	34.9	
I eat more because I have nothing else to do	19.7	20.1		19.4	20.5		19.1	20.0	
**Since schools are closed**			0.064			0.017			<0.001
I play more with my family	21.7	24.9		23.0	22.9		33.2	20.6	
I fight more with my brothers/sisters	15.5	13.2		14.9	14.2		22.5	12.9	
I fight more with my parents	21.1	17.7		21.7	16.7		16.8	20.5	
I speak more with my family	41.7	44.2		40.5	46.3		27.5	46.0	
**Ever since schools are closed relationships with your parents**			0.004			0.132			0.058
They have improved	24.5	19.0		21.4	24.3		22.7	22.4	
They have gotten worse	9.7	8.2		9.9	7.9		12.3	8.5	
They are always the same	65.8	72.8		68.7	67.8		65.0	69.1	
**Ever since schools are closed and the authorities say they are decreasing outings and contacts with others. Are you missing something?**			<0.001			0.064			<0.001
No	2.4	4.8		3.3	3.4		4.3	3.1	
Yes, my friends	49.6	47.2		47.4	50.8		52.4	47.9	
Yes, sports activity	7.9	16.4		12.3	9.0		20.6	8.9	
Yes, the festive occasions	24.9	18.0		21.5	23.8		9.1	25.3	
Other	15.3	13.6		15.6	13.1		13.6	14.8	
**Do you ever talk about COVID-19 with your friends?**			<0.001			<0.001			<0.001
Never	2.8	12.5		7.4	4.8		15.2	4.4	
Very often	16.0	6.1		10.2	15.9		5.3	13.8	
Sometimes	43.8	56.5		50.5	45.1		60.7	45.8	
Often	37.5	24.9		31.9	34.2		18.7	35.9	
**Would you like to be able to discuss/talk more about the COVID-19 virus?**			<0.001			<0.001			0.005
No	45.0	55.4		52.3	43.2		56.9	47.1	
Yes, to adopt behavior	10.0	6.5		7.4	10.7		7.8	8.9	
Yes, for knowledge	29.8	26.9		27.5	30.8		21.7	30.3	
Yes, for reassurance	12.3	8.1		9.2	13.2		10.2	10.8	
Other	2.9	3.1		3.5	2.2		3.5	48.9	
**Who would you like to talk to about COVID-19?**			<0.001			0.01			<0.001
Parents	17.7	24.0		19.6	20.8		35.3	16.7	
Teachers	17.8	13.2		17.1	14.3		13.4	16.7	
With a doctor	51.5	44.7		46.9	52.5		31.6	52.8	
With nobody	1.3	1.7		5.1	1.4		4.8	4.0	
Other	11.7	16.4		11.3	11.0		15.0	9.8	
**Ever since you have been home, have you ever had a fever for fear of COVID-19?**			0.078			<0.001			0.111
Twice a week	2.4	1.4		2.6	1.2		1.6	2.1	
No	74.6	79.8		72.3	83.5		82.1	75.3	
Occasionally	15.9	12.4		17.9	9.0		11.8	15.2	
Yes, several times a day	3.0	2.1		3.2	1.8		1.3	3.0	
Yes, once a day	3.2	3.5		3.3	3.4		2.4	3.5	
Three times a week	1.0	0.8		0.8	1.2		0.8	0.9	
**Since when have you been home from school, have you been visited for COVID-19?**			0.078			<0.001			0.408
No, but I wished because I had a fever	1.8	2.4		2.6	0.9		1.3	2.13	
No, but I wished because I was afraid of having symptoms	4.9	2.7		4.3	3.6		3.5	4.2	
No, there was no need	91.9	93.7		90.9	99.9		94.1	92.25	
Yes, I had a fever and other COVID-19 symptoms	0.7	0.9		1.2	0.1		0.8	0.77	
Yes, my family members have been hospitalized	0.7	0.1		0.8	0.0		0.0	0.59	
Yes, I am regularly followed to the hospital	0.1	0.1		0.2	0.0		0.3	0.1	
**Would you like to make a swab to find out if you have COVID-19?**			0.014			0.241			0.035
I've already done it	0.5	0.9		0.9	0.3		0.8	0.6	
No	49.5	55.3		51.2	52.3		57.5	50.4	
Yes	50.0	43.8		47.9	47.4		41.7	49.0	
**Would you like to take a blood sample to find out if you have had COVID-19?**			<0.001			0.011			<0.001
I've already done it	0.8	0.9		1.1	0.4		1.0	0.8	
No	49.0	58.0		50.1	56.1		65.0	49.5	
Yesi	50.3	41.1		48.8	43.5		34.0	49.7	

No differences between groups were found for the question about “washing my hands more than before” except for by age group: 87.4% of the 11–13-year group and 82.5% of the 14–19-year group reported washing their hand more than before (*p* < 0.01). Differences were found for the habit of using the surgical mask between males and females and the two age groups, with males and older adolescents using face masks less than females and younger adolescents (*p* < 0.001). However, no differences were evidenced among Northern and Southern students.

### Psychological Impact of Covid-19

Table 5 shows the psychological impact of school closures due to the COVID-19 lockdown in the study population. As shown, problems were evidenced in all the study groups, although with greater frequency among females, and students living in the regions with greater incidence of COVID-19. This was clearly evidenced when fear of Covid-19 was considered. This was reported in 85.4% of females and in 63.5% of males (*p* < 0.001). Moreover, fear of losing grandparents was more common in the northern regions (33.6%) than in the southern regions (22%) (*p* < 0.001) and in the 11–13-year age group (36.6%) than the 14–19-year age group (27.6%; *p* < 0.001). The feeling of sadness was significantly more frequent in females (84%) than in males (68.2%) (*p* < 0.001) and in the 14–19-year age group (79.2%) (*p* < 0.001). Loneliness was identified as the primary cause of sadness in all groups (ranging from 32.3% of males to 42.5% of females). Missing the school community was a significantly more common cause of sadness in girls (26.5% vs. 16.8%; *p* < 0.001), in the southern regions (26.45% vs. 20.2%; *p* < 0.01) and in the 14–19-year-old group (24.2% vs. 14.7%; *p* < 0.001). In addition, 48.7% of females reported crying during the day (13.4% in males; *p* < 0.001). A sense of fatigue was more frequent in females than in males (*p* < 0.05) and in the 14–19-year age group than in the 11–13-year age group (*p* < 0.001). No differences were found between northern and southern Italy. Similarly, a sense of agitation was more frequent in females than males (61.9% vs. 42.5%; *p* < 0.001). The ability to see classmates was the aspect that was most commonly missed by adolescents during school closures regardless of gender, age group, or geographic location.

**Table 5:** Questionnaire results about lifestyles during school closure because of COVID-19 lockdown in the study population according to gender, area of residence, and age.

	**Females**	**Males**	***p*-value**	**North**	**South**	***p*-value**	**% 11–13**	**% 14–19**	***p*-value**
**Do you make school online?**			0.454			0.328			0.119
No	1.2	0.7		0.9	1.2		1.3	0.9	
Yes, I'm not satisfied	34.4	33.4		35.1	32.2		29.7	35.0	
Yes, I am satisfied	64.5	66.0		64.0	66.7		69	64.1	
**Do you go out sometimes?**			<0.001			<0.001			0.001
No	77.8	69.2		69.5	83.1		69.8	75.7	
Yes, 2–3 days a week	5.3	8.0		8.1	3.4		8.8	5.7	
Yes, sometimes	5.1	11.1		9.6	3.5		11.2	6.5	
Yes, once a week	11.8	11.7		12.8	10.1		10.2	12.1	
**Do you meet some friends?**			0.06			0.048			0.094
No	94.06	91.66		92.09	94.96		91.2	93.6	
Yes, every 2–3 days	2	2.22		2.64	1.16		3.5	1.8	
Yes, every day	1.62	3.39		2.4	2.07		3.2	2.1	
Yes, once a week	2.31	2.74		2.87	1.81		2.1	2.5	
**Since when are schools closed have you been spending more time on video games?**			<0.001			0.025			<0.001
No	56.8	24.0		42.6	48.3		34.8	46.9	
Yes, 1 h more	5.5	16.4		10.9	7.4		16.8	7.9	
Yes, 1 to 3 more hours	5.1	14.3		8.8	8.0		10.2	8.2	
Yes, more than 3 h	3.2	11.2		6.0	6.6		6.1	6.2	
Certainly more but I don't know how to quantify	29.5	33.6		31.8	29.7		32.1	30.8	
**Do you wash your hands more than before?**			0.498			0.843			0.020
No	16.2	17.3		16.7	16.4		12.6	17.5	
Yes	83.8	82.7		83.3	83.6		87.4	82.5	
**Do you have a surgical mask?**			0.001			0.555			<0.001
No	14.3	19.2		16.1	16.3		20.3	15.2	
Yes, and I use it regularly if I leave the house	50.7	42.8		48.6	46.3		37.7	50.0	
Yes, but I've never worn it	35.0	38.1		35.4	37.5		42.0	34.8	

Since schools had been closed, males reported sleeping more than before (46.8%), while 43.1% of females stated they slept less than before (*p* < 0.001). An analysis of these data by age group showed that the younger students (11–13 years) were more likely than older students to report sleeping more than before school closures (*p* < 0.001). Difficulty falling asleep was the most frequent reason for sleeping less among the groups. A lack of interaction with schoolmates/friends was the main reason for displeasure in all groups, with significant differences between males and females (70.9% vs. 58.1%; *p* < 0.001), between students in the northern and southern regions (65.4% vs. 58.8%; *p* < 0.01) and between age groups (11–13 years, 69.5%; 14–19 years, 61.4%; *p* < 0.01). A lack of sports activity was significantly more frequent in the 11–13-year age group (20.6% vs. 8.9%) and in males (16.4% vs. 7.9%; *p* < 0.001).

Most of the subjects reported eating more during the quarantine period, with a statistically significant difference between males and females (48.8 vs. 55.9%; *p* < 0.01). Of particular interest were the data concerning the relationship with parents: 68.4% of the sample declared that their relationships had remained the same, 24.5% of the females reported that their relationships had improved (compared to 19.0% of the males; *p* < 0.01). In addition, most of the teenagers said that they spoke more with their parents (42.6%), with a higher percentage of students in norther than southern Italy (46.3 vs. 40.5%; *p* < 0.05) and a higher percentage of students in the 14–19-year age group than the 11–13-year age group (46.0 vs. 27.5%; *p* < 0.01) reporting this change.

When asked if they talked about Covid-19 with friends, 50.1% of the students from the southern regions reported doing so often or very often (vs. 42.1% of the students from the northern regions). The responses to the question asking who students would like to talk about the virus, similar percentages were found between regions: 52.5% of southern students said they would like to speak with a doctor (vs. 46.9% of northern students).

To understand the degree of fear that teenagers experienced, we asked them whether they had taken their temperature during the quarantine and, if so, how many times. The resulting data were: 6.2% of the females and 5.6% of the males said they had taken their temperature once or several times a day. However, approximately 52% of the sample replied that they did not want to undergo a swab test (51.7%) or provide a blood sample (52.3%) to determine if they had–or had previously had–the virus.

A global evaluation of all these findings through the feeling index showed that school closure impact on mental health was slightly greater in females and in subjects living in the Northern Regions possibly due to the greater clinical relevance of COVID-19 in these areas and the greater personal experience with a relative or an acquaintance affected by this disease. The multivariate regression analysis showed that male sex was a protective factor against negative feelings (*p* < 0.01), leading to a decrease of 0.63 points in the total negative feelings index. Having a family member or an acquaintance with COVID-19 increased the negative feelings index by 0.1 points (*p* < 0.05).

## Discussion

In this study, most students who completed the administered questionnaire showed good knowledge of the epidemiological, clinical and therapeutic characteristics of COVID-19, as well as of disease risks, regardless of sex, age, location, and type of school. Only the source of information was slightly different in younger students than the older ones. Younger students preferred to consult with parents, while older students drew most of their knowledge on COVID-19 from mass media, likely due to the increased autonomy of adolescents with age (14). Teenagers' good knowledge of COVID-19 even in Italian Regions where diffusion of COVID-19 was relatively low is not surprising, as the outbreak of the pandemic has had profound impact on all aspects of people's everyday lives, mainly because of the very restrictive preventive measures put in place by most governments, including the closure of factories, commercial activities and schools (14). All these findings have had very wide resonance in the mass media and it is highly likely that they have been repeatedly discussed within families mainly because of the organization of the distance learning (15). Adolescents were therefore widely informed about COVID-19 and the evolution of the pandemic. Moreover, with school closures and the need to be compliant with recommendations by authorities, teenagers in most countries have personally experienced several conditions that have made them aware of the medical, social, and economic consequences of COVID-19 (16).

Modifications of lifestyle were also clearly evidenced in the population that participated in the study, but with slight differences according to sex, age, and location. In general, they seem related to both school closure that prevented socialization and the good knowledge of the danger of COVID-19 which led to a good acceptance of the health authority recommendations for COVID-19 spread containment (17, 18). However, adherence to some of these, such as frequent hand washing and use of masks, was greater among females than among males. This seems to confirm what has been repeatedly reported in more complex studies, i.e., that girls are more compliant than boys with the requests and demands of parents, teachers, and other authorities (19).

This study shows that school closures because of the COVID-19 lockdown have several negative psychological consequences among adolescents, even when they have not been infected with SARS-CoV-2. As expected, negative feeling was more common among subject living in northern regions where children and adolescents have had more frequent opportunities to see directly the clinical significance of COVID-19. Impaired mental health in conditions such as those due to COVID-19 pandemic has been previously shown by several studies evidencing that home confinement of children and adolescents cause uncertainty and anxiety which is ascribable to disruption in education, physical activities and socialization (20). Results quite similar to those of this study have been recently reported by Zhou et al. (12), who surveyed 8,079 Chinese adolescents during the COVID-19 outbreak and reported symptoms of depression, anxiety and combined depression and anxiety in 43, 37, and 31% of the adolescents, respectively. As found in this study, in the previous study psychological symptoms were significantly more common among females. The prevalence of mental disorders among females is not surprising because, independent of the pandemic outbreak, anxiety, and depressive symptoms are generally more common among women, even in the adolescent period (13). On the other hand, questions included in the questionnaire were focused on the evidence of internalizing symptoms that are generally more common among females and not on externalizing problems such as rule–breaking actions and aggressive behavior, that are more frequently diagnosed in males (19). However, the incidence of psychological problems in the studied population was also significant among males, highlighting that all adolescents may suffer from psychological problems when facing unexpected dangerous events. In addition, adolescence is a crucial period of life characterized by multiple physical, emotional, and social changes that expose the individual to several risk factors for the development of mental health problems. Up to 20% of adolescents suffer from mental disabilities, mainly depression and anxiety (21, 22), and it is not surprising that the emergence of a stressful event such as a pandemic outbreak would make these conditions even more evident, particularly when the event is associated with school closures that reduce social connections and access to mental health services. A study carried out in the UK (13) involving more than 2100 adolescents with mental problems showed that most of them (83%) had experienced a worsening of their mental conditions. More than a quarter of enrolled subjects reported that they were no longer able to access mental health support.

At the beginning of the pandemic, considering previous experience with influenza (23), children and adolescents were considered among the major populations responsible for SARS-CoV-2 transmission; thus, to reduce risks, any type of school was closed in most of the countries. However, this decision was thoroughly discussed by many experts for several reasons (24). Although the actual role of pediatric patients as a cause of SARS-CoV-2 diffusion was not precisely defined, the possibility could not be excluded that adolescents could have a significant role in this regard (25); however, school closures could cause a number of social, mental, economic and nutritional problems that are even greater than those due to the infection. Our data on the psychological impact of school closures in children and adolescents because of COVID-19 lockdown highlight that school closures should be avoided and that school system infrastructure, the number of employees and their education on infectious disease prevention should be improved to ensure safe school attendance.

This study has several limitations. The sample size was small, and it is possible that it may not represent the Italian population of older children and adolescents due to the recruitment method and because of differential internet access. The use of the online tool limits access to persons who can use this technology, whereas in Italy, particularly in the Southern Regions, many students do not have adequate connection and personal computer. Moreover, collected data only refer to the first period of COVID-19 pandemic and it is possible that after a longer lockdown the impact of the pandemic could have been different. Finally, the study does not define whether lifestyle and mental health problems are simply due to school closure or the hard lockdown simultaneously decided by authorities.

## Conclusions

Data collected with this study can be useful to show that school closures can cause substantial emotional uncertainty among adolescents and subjects living in geographic areas with high incidence of COVID-19 cases. This indicates the relevance of a strict monitoring of mental health of older children and adolescents during school closure and the need for schools to be reopened as soon as possible accordingly with the evolution of the pandemic.

## Data Availability Statement

The raw data supporting the conclusions of this article will be made available by the authors, without undue reservation.

## Ethics Statement

The studies involving human participants were reviewed and approved by University of Parma, Parma, Italy. Written informed consent to participate in this study was provided by the participants' legal guardian/next of kin.

## Author Contributions

SE designed the study, participated in data analysis, and wrote the first draft of the manuscript. NG participated in enrolment and data entry. AS evaluated the psychological results. CN and AA performed data entry and statistical analysis. PM participated in data entry and performed the literature review. NC gave a substantial scientific contribution. NP co-wrote the first draft of the manuscript and made substantial scientific contributions. All authors approved the final version of the manuscript.

## Conflict of Interest

The authors declare that the research was conducted in the absence of any commercial or financial relationships that could be construed as a potential conflict of interest.
